# Histopathological changes in parotid gland following submandibular gland failure: an experimental animal study^☆^

**DOI:** 10.1016/j.bjorl.2018.03.013

**Published:** 2018-04-24

**Authors:** Yavuz Sultan Selim Yıldırım, Irfan Kaygusuz, Ibrahim Hanifi Ozercan, Hasan Cetiner, Oner Sakallioglu, Abdulvahap Akyigit, Sertac Duzer

**Affiliations:** a Elazig Education and Research Hospital, Department of Otorhinolaryngology, Elazig, Turkey; b University of Firat, School of Medicine, Department of Otorhinolaryngology, Elazig, Turkey

**Keywords:** Submandibular, Parotid, Submandibular gland failure, Histopathology, Submandibular, Parótida, Insuficiência da glândula submandibular, Histopatologia

## Abstract

**Introduction:**

Submandibular glands are exposed to many effects due to diseases and therapeutic interventions. A study evaluating the effect of submandibular gland dysfunction on the parotid gland has not been presented in the literature.

**Objective:**

The aim of this study was to evaluate the histopathological changes in the parotid gland following submandibular gland failure.

**Methods:**

Three groups of seven randomly selected female New Zealand rabbits weighing 2500–3000 g were studied. Unilateral and bilateral submandibular glands were removed in Groups 1 and 2, respectively. No procedure was performed in Group III, the control group. The parotid glands were removed 30 days later. Histological parameters were evaluated and graded between 0 (none) and 3 (severe). Differences between groups were compared using the Mann–Whitney *U* test.

**Results:**

Mean mucus accumulation in acinar cells was 2.57 ± 0.53 and 1.71 ± 0.75 in Groups 1 and 2, respectively (*p* < 0.05). This value was 0.57 ± 0.53 in Group 3, which was significantly lower than in Groups 1 and 2 (*p* < 0.05). Mean dilatation of the intercalated ducts’ lumen was 1.28 ± 0.48 and 1.57 ± 0.53 in Groups 1 and 2, respectively (*p* > 0.05). This value was 0.28 ± 0.48 in Group 3, which was significantly lower than in Groups 1 and 2 (*p* < 0.05). Mean mucus accumulation in the intercalated ducts’ lumen was 2.00 ± 0.81 and 1.00 ± 0.57 in Groups 2 and 3, respectively (*p* < 0.05).

**Conclusion:**

The findings of this study indicate that only 1 month after submandibular gland failure, the parotid glands exhibit significant changes.

## Resumo

**Introdução:**

As glândulas submandibulares estão expostas a muitos efeitos causados por doenças e intervenções terapêuticas. Estudos que avaliam o efeito da disfunção da glândula submandibular na glândula parótida ainda não foram reportados na literatura.

**Objetivo:**

O objetivo deste estudo foi avaliar as alterações histopatológicas na glândula parótida após insuficiência da glândula submandibular.

**Método:**

Três grupos de sete coelhas fêmeas da raça Nova Zelândia, selecionadas aleatoriamente, pesando entre 2.500 e 3.000 gramas foram estudadas. As glândulas submandibulares unilaterais e bilaterais foram removidas nos Grupos 1 e 2, respectivamente. Nenhum procedimento foi realizado no Grupo III, o grupo controle. As glândulas parótidas foram removidas 30 dias depois. Os parâmetros histológicos foram avaliados e classificados entre 0 (nenhum) e 3 (grave). As diferenças entre os grupos foram comparadas usando o teste U de Mann-Whitney.

**Resultados:**

O acúmulo médio de muco nas células acinares foi de 2,57 ± 0,53 e 1,71 ± 0,75 nos Grupos 1 e 2, respectivamente (*p* < 0,05). Esse valor foi de 0,57 ± 0,53 no Grupo 3, significativamente menor do que nos Grupos 1 e 2 (*p* < 0,05). A dilatação média do lúmen dos dutos intercalados foi de 1,28 ± 0,48 e 1,57 ± 0,53 nos Grupos 1 e 2, respectivamente (*p* > 0,05). Esse valor foi de 0,28 ± 0,48 no Grupo 3, significativamente menor do que nos Grupos 1 e 2 (*p* < 0,05). O acúmulo médio de muco no lúmen dos dutos intercalados foi 2,00 ± 0,81 e 1,00 ± 0,57 nos Grupos 2 e 3, respectivamente (*p* < 0,05).

**Conclusão:**

Os achados deste estudo indicam que apenas um mês após a insuficiência da glândula submandibular, as glândulas parótidas apresentam alterações significativas.

## Introduction

Three pairs of major salivary glands are positioned around the oral cavity: the sublingual glands under the tongue, the submandibular glands under the floor of the mouth, and the parotid glands in the posterior aspect of the mouth at the retromandibular fossae.^1^

The major salivary glands are exposed to a number of factors which leads to loss of function. Some of these factors such as botulinum toxin injection, gland excision, and gland transfer are intended for therapeutic purposes.^2^, ^3^, ^4^ Salivary gland stones, radiotherapy applied to the head and neck region, and salivary gland trauma cause significant dysfunctions.^5^, ^6^, ^7^ In the lack of a study evaluating the effect of any major salivary gland (or glands) dysfunction on the other salivary glands, our aim was to evaluate the histopathological changes of the parotid gland in a rabbit model of impaired submandibular gland function.

## Methods

### Study design and animals

This study was performed on 21 female New Zealand rabbits (2500–3000 g) in the Center for Experimental Research of the University of Firat, after obtaining approval from the Ethics Board of the School of Medicine of the University of Firat (Number of Document: 142563/14). The animals were randomly divided into three groups, with seven in each group:

• Group I: Unilateral submandibular glands were resected, and bilateral parotid glands were removed 30 days later for histopathological examination.
• Group II: Bilateral submandibular glands were resected, and bilateral parotid glands were removed 30 days later for histopathological examination.
• Group III (control group): No surgical intervention was performed on the submandibular glands, and bilateral parotid glands were removed for histopathological examination.

### Surgical procedure

**Figure 1:**
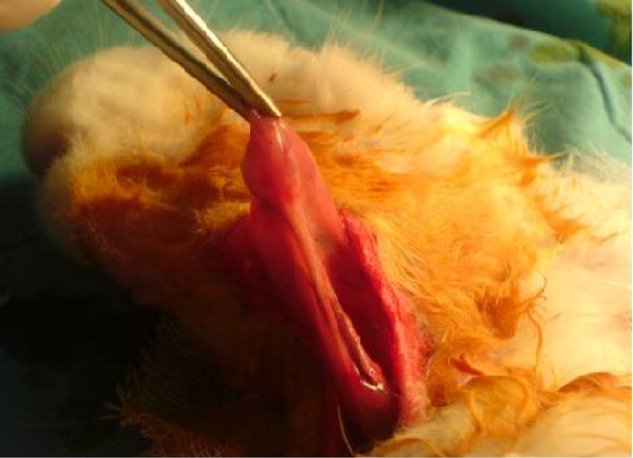
Submandibular gland excision.

The same surgical procedure was performed in all Group I and II rabbits. The animals were anesthetized using 10 mg/kg xylazine hydrochloride (Rompun^®^; Bayer AG, Germany) and 50 mg/kg ketamine hydrochloride (Ketalar^®^; Eczacibasi Ilac, Turkey). A 3 cm long horizontal incision was created 1 cm under the corpus of the mandible. The submandibular glands were exposed after elevating skin and subcutaneous flaps (Fig. 1). Unilateral and bilateral submandibular glands were excised from the animals in Groups I and II, respectively. Prophylactic 20–40 mg/kg cephazolin sodium (Sefazol Flk^®^; Mustafa Nevzat, Turkey) was administered 1 h before and 1 h after surgery. All animals were followed for 30 days after the surgical procedure.

The rabbits were anesthetized again using 10 mg/kg xylazine hydrochloride and 50 mg/kg ketamine hydrochloride on the 30th postoperative day. An incision approximately 3 cm in length was made in the region of the parotid gland. The parotid gland was exposed by elevating skin and subcutaneous flaps and then excised. The same surgical procedure was performed on the contralateral parotid gland.

### Specimen preparation

The parotid glands were fixed in 10% glutaraldehyde for 4–6 h. Fixated specimens were gradually dehydrated in ethanol after being maintained in 1% osmium tetroxide for 0.5 hour and then placed in Epon. Ultratome III glass knives (Shandan Finesse, United Kingdom) were used to obtain 1.5 μm thick sections. The sections were stained in hematoxylin and eosin and examined at 40×, 100×, 200×, and 1000× magnification using a light microscope (Olympus, BX51, Japan).

### Specimen evaluation

Mucus accumulation in acinar cells, dilatation of intercalated ducts’ lumen, mucus accumulation in the lumen of intercalated ducts, cytoplasmic granule accumulation, and myoepithelial cell count were assessed using Eyepieces graticule (an ocular micrometer with 100 equal squares measuring 1 × 1 mm each) mounted on an Olympus light microscope.

From each rabbit, four sections were collected. Within each section, four distinct areas were examined at four magnifications (40×, 100×, 200×, and 1000×) and graded as follows: none = 0, mild = 1, intermediate = 2, and severe = 3. The mean score was calculated using all four examined areas of all four sections from each rabbit.

### Statistical analysis

A database was created using the histopathological data, which was statistically analyzed using SPSS 11.5 package program (SPSS Inc, ABD). Values of *p* less than 0.05 were accepted as statistically significant. The variables had a non-normal distribution since each group was composed of seven rabbits and the total number of rabbits was 21; thus, non-parametric tests were used in the statistical analyses. The Mann–Whitney *U* test was used to compare groups.

## Results

Mean mucus accumulation in acinar cells was 2.57 ± 0.53 in Group 1 and 1.71 ± 0.75 in Group 2 (*p* < 0.05). This value was 0.57 ± 0.53 in Group 3, which was significantly lower than in Groups 1 and 2 (*p* < 0.05). Mean dilatation of the intercalated ducts’ lumen was 1.28 ± 0.48 in Group 1 and 1.57 ± 0.53 in Group 2 (*p* > 0.05). This value was 0.28 ± 0.48 in Group 3, which was significantly lower than in Groups 1 and 2 (*p* < 0.05). Mean mucus accumulation in the intercalated ducts’ lumen was 2.00 ± 0.81 in Group 2 and 1.00 ± 0.57 in Group 3 (*p* < 0.05). This value was 1.42 ± 0.53 in Group 1, which was not significantly different from the intercalated ducts mucus accumulation in either of the other two groups. There were no significant differences between groups in terms of cytoplasmic granule accumulation or number of myoepithelial cells (Table 1).

**Table 1:** Means and standard deviation values of experiment (Group I–II) and control group (Group III) data.

	Group I	Group II	Group III
Mucus accumulation in acinar cells	2.57 ± 0.53	1.71 ± 0.75	0.57 ± 0.53
Mucus accumulation in the intercalated ducts’ lumen	1.42 ± 0.53	2.00 ± 0.81	1.00 ± 0.57
Cytoplasmic granule accumulation	1.71 ± 0.48	1.28 ± 0.75	1.28 ± 0.95
Dilatation in intercalated ducts’ lumen	1.28 ± 0.48	1.57 ± 0.53	0.28 ± 0.48
Myoepithelial cell count	1.71 ± 0.75	1.71 ± 0.75	1.85 ± 0.69

## Discussion

Secretions are produced in salivary glands by structures composed of mucous and serous cells called acini. Each acinus opens into an intercalated duct, which in turn drains into a striated duct. Myoepithelial cells are found in the basal laminae of the epithelium near the acinus and in the basal lamina of the canal epithelium. Acini and intercalated ducts are surrounded by myoepithelial cells.^8^, ^9^ The parotid gland is composed of serous acinar cells containing intense intracytoplasmic translucent granules that produce a pure serous secretion.^10^

**Figure 2:**
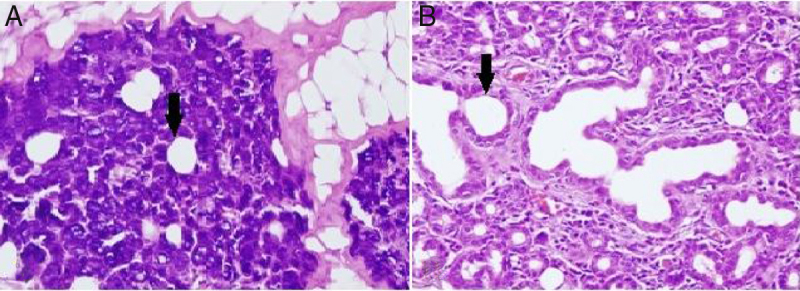
(A) Dilatation in intercalated ducts’ lumen in parotid gland acinar cells of the unilateral submandibular gland excision group (200× light microscope H&E). (B) Dilatation in intercalated ducts’ lumen in parotid gland acinar cells of the bilateral submandibular gland excision group (200× light microscope H&E). ↓, lumen of intercalate duct.

Alterations in the histological structure of the parotid gland are seen in tumors, inflammatory conditions, systemic diseases, and following radiation treatment; thus, the secretory function of the gland deteriorates.^11^ In the present study, loss of submandibular gland function was shown to cause a proportional dilatation of the intercalated ducts’ lumen (Fig. 2). Similar to our study, intercalated ducts’ lumen dilatation is also seen in sialolithiasis and excessive alcohol consumption.^12^, ^13^ Squamous metaplasia in the epithelium, intermediate to severe chronic inflammation, and varying degrees of acinar destruction were reported in biopsy material obtained from a gland with sialolithiasis.^12^ These extra findings in sialolithiasis are not presented in our own work which is probably due to the fact that the time of study limited as little as a month. In contrast to our study, intracytoplasmic granule accumulation is an important finding of excessive alcohol consumption.^13^

**Figure 3:**
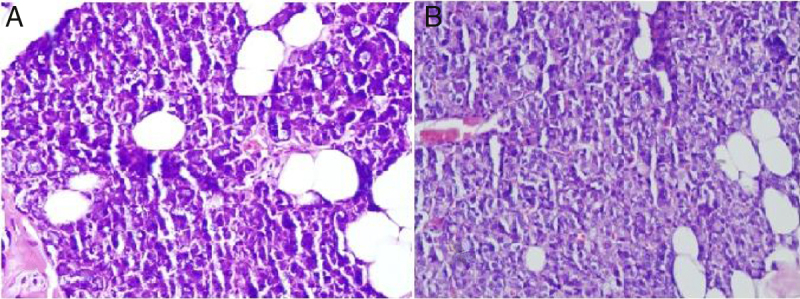
(A) Mucus accumulation in parotid gland acinar cells of the unilateral submandibular gland excision group (400× light microscope H&E). (B) Mucus accumulation in parotid gland acinar cells of the bilateral submandibular gland excision group (400× light microscope H&E).

The main ductal ectasia of salivary gland is one of the histopathological findings of Sjögren's syndrome and infectious diseases.^14^, ^15^ Also, interstitial fibrosis and metaplastic changes are the mainstays of radiotherapy-related gland damage.^16^ While these findings are not observed in our study, mucus accumulation is another important finding of the histopathological examination. Mucus accumulation in acinar cells was greater in animals with unilateral and bilateral submandibular gland excision compared to the control group, and there was also a statistically significant difference between the unilateral and bilateral groups (Fig. 3). Mucus accumulation in the lumen of intercalated ducts was also greater in animals with bilateral submandibular gland excision compared to the control group.

## Conclusion

Unilateral or bilateral excision of the submandibular gland results in histopathological changes in the parotid gland after a short period of time. When the histopathological changes in other diseases affecting the salivary gland are considered, the dilatation of the intercalated ducts’ lumen in our study was consistent with findings in salivary gland diseases related to infectious and overuse of alcohol. The cause of histopathological changes in the parotid glands after a short time (1 month) might be explained by the parotid glands being overworked, as they must compensate for the large workload previously shared by the submandibular gland.

The present study can be considered a good model for evaluating the short-term effects of decreased submandibular gland function on the parotid gland. Nevertheless, additional studies are required to evaluate long-term effects such as metaplasia and etc.

## Conflicts of interest

The authors declare no conflicts of interest.

## References

[1] Tucker A.S. (2007). Salivary gland development. Semin Cell Dev Biol.

[2] Jongerius P.H., Joosten F., Hoogen F.J.A., Gabreels F.J., Rotteveel J.J. (2003). The treatment of drooling by ultra-sound guided intra-glandular injections of botulinum toxin-A into the salivary glands. Laryngoscope.

[3] Stern Y., Feinmesser R., Collins M., Shott S.R., Cotton R.T. (2002). Bilateral submandibular gland excision with parotid duct ligation for treatment of sialorrhea in children: long-term results. Arch Otolaryngol Head Neck Surg.

[4] Naresh J.H.A., Seikaly H., Mcgaw T., Coulter L. (2000). Submandibular salivary gland transfer prevents radiation-induced xerostomia. Int J Radiat Oncol Biol Phys.

[5] Carta F., Farneti P., Cantore S., Macri G., Chuchueva N., Cuffaro L. (2017). Sialendoscopy for salivary stones: principles, technical skills and therapeutic experience. Acta Otorhinolaryngol Ital.

[6] Lovelace T.L., Fox N.F., Sood A.J., Nguyen S.A., Day T.A. (2014). Management of radiotherapy-induced salivary hypofunction and consequent xerostomia in patients with oral or head and neck cancer: meta-analysis and literature review. Oral Surg Oral Med Oral Pathol Oral Radiol Endod.

[7] Haller J.R. (1999). Trauma to the salivary glands. Otolaryngol Clin North Am.

[8] Eisbruch A., Rhodus N., Rosenthal D., Murphy B., Rasch C., Sonis S. (2003). How should we measure and report radiotherapy-induced xerostomia?. Semin Radiat Oncol.

[9] Radfar L., Sirois D.A. (2003). Structural and functional injury in minipig salivary glands following fractionated exposure to 70 Gy of ionizing radiation: an animal model for human radiation-induced salivary gland injury. Oral Surg Oral Med Oral Pathol Oral Radiol Endod.

[10] Henriksson R., Frojd O., Gustafsson H., Johansson S., Yi-Qing C., Franzén L. (1994). Increase in mast cells and hyaluronic acid correlates to radiation-induced damage and loss of serous acinar cells in salivary glands: the parotid and submandibular glands differ in radiation sensitivity. Br J Cancer.

[11] Croce A., D’agostıno L., Moretti A., Augurio A. (2008). Parotid surgery in patients over seventy-five years old. Acta Otorhinolaryngol Ital.

[12] Andretta M., Tregnaghi A., Prosenikliev, Staffieri A. (2005). Current opinions in sialolithiasis diagnosis and treatment. Acta Otorhinolaryngol Ital.

[13] Carranza M., Gallizi M. (2005). Structural and morphometrical study in glandular parenchyma from alcoholic sialosis. J Oral Pathol Med.

[14] Eroschenko V.P., Mariano S.H. (2008).

[15] Bone R.C. (1985). Sjogren syndrome, a persistent clinical problem. Laryngoscope.

[16] Radfar L., Cheng S.C. (2011). Assessment of post-radiotherapy salivary glands. Br J Radiol.

